# Supplementary material for: A Zebrafish Mutant in the Extracellular Matrix Protein Gene efemp1 as a Model for Spinal Osteoarthritis
Source: Animals (Basel). 2023 Dec 24;14(1):74. doi: 10.3390/ani14010074 (PMC10778253; doi:10.3390/ani14010074)

animals MDPI

Figure S1. No significant effect on bone mineralization in efemp1−/− mutants at 5 dpf compared to WT. (A) Ventral view of alizarin red stained WT and efemp1−/− larvae at 5 dpf. The blue arrowheads point to the skeletal elements: branchiostegal ray1 (br1), ceratohyal (ch), dentary (d), entopterygoid (en), hyomandibular (hm), maxillary (m), opercle (op) and parasphenoid (p). (B) Fraction (%) of indi-viduals presenting a high (maroon), normal/intermediate (red), reduced/ low (light red), or absent (white) level of bone mineralization in the different bone elements in WT and efemp1−/− fish at 5dpf. (WT n = 24, efemp1−/− n = 15).

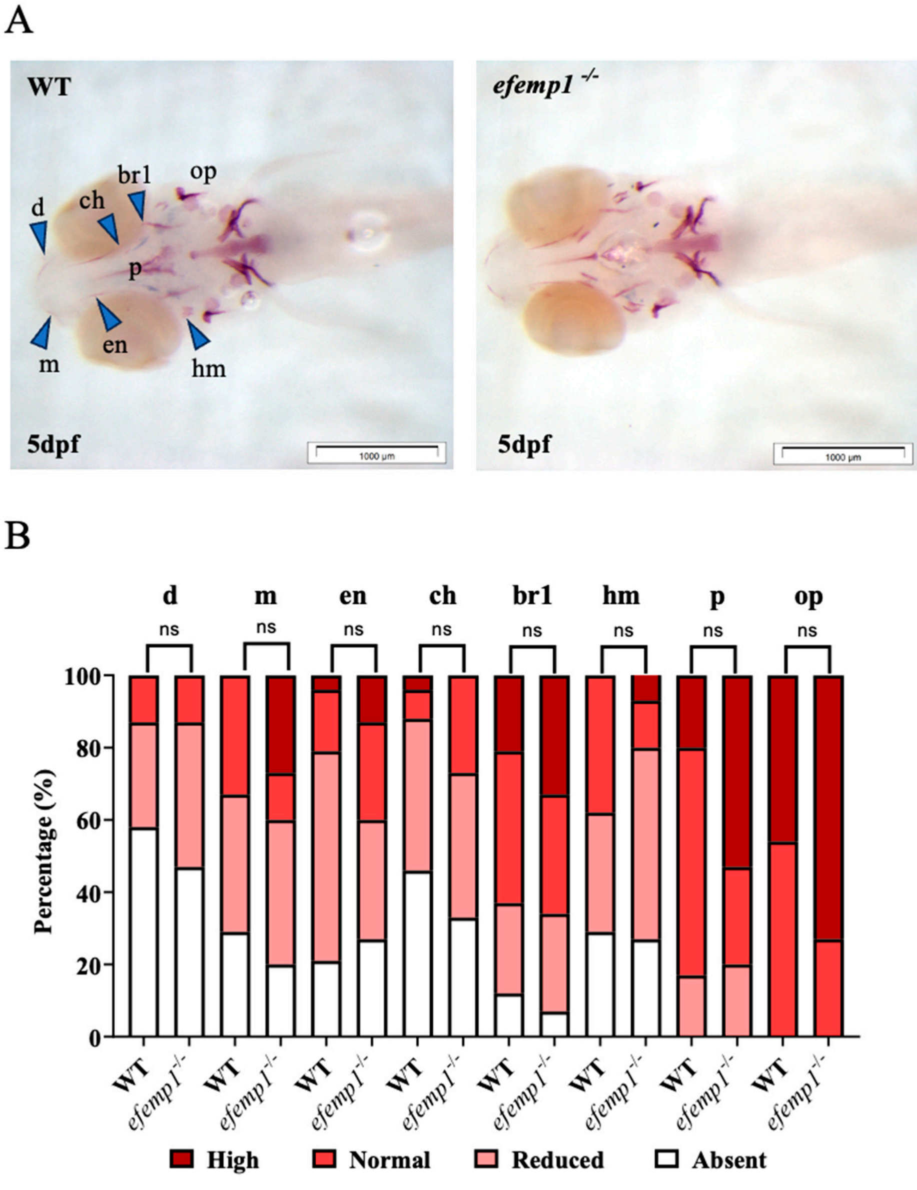

Supplement: Supplementary file 1 [file animals-14-00074-s001.zip › animals-2699407-supplementary.pdf]
